# Application of TyG index and carotid ultrasound parameters in the prediction of ischemic stroke

**DOI:** 10.3389/fendo.2025.1481676

**Published:** 2025-03-03

**Authors:** Huimin Guo, Sen Wang, Haizheng Wang, Li Feng

**Affiliations:** ^1^ Department of Medical Ultrasound, The First Affiliated Hospital of Shandong First Medical University and Shandong Provincial Qian Foshan Hospital, Shandong Medicine and Health Key Laboratory of Abdominal Medical Imaging, Jinan, Shandong, China; ^2^ School of Clinical Medicine, Jining Medical University, Jining, Shandong, China

**Keywords:** carotid ultrasound parameters, TyG index, ischemic stroke(IS), prediction model, insulin resistance (IR)

## Abstract

**Objective:**

The triglyceride - glucose (TyG) index has been confirmed as an independent risk factor for ischemic stroke (IS) in numerous studies. In terms of the role of carotid ultrasound in the risk assessment of IS, the focus has shifted from merely concentrating on the degree of stenosis to paying more attention to the status of carotid plaques. However, there are limited studies on combining clinical indicators such as the TyG index with carotid ultrasound parameters to assess the risk of IS. Through a retrospective study, we aim to explore the role of combining these two types of indicators in the risk assessment of IS

**Methods:**

This study included 145 patients with IS and 99 no ischemic stroke (NIS) patients diagnosed by magnetic resonance imaging (MRI) from January 2020 to June 2024. The TyG index was calculated as ln [fasting triglyceride (mg/dL) × fasting blood glucose (mg/dL)/2]. The carotid ultrasound parameters integrated were as follows: the presence or absence of carotid plaques, the location of the largest carotid plaque, carotid intima - media thickness (CIMT), the lengthness and thickness diameters of the largest carotid plaque, and the degree of carotid stenosis. Univariate (multivariate) logistic regression analysis, ROC curve analysis, etc. were conducted on the data using SPSS 26 and MATLAB Online. These were aimed at assessing the effectiveness of integrating clinical indicators with carotid ultrasound parameters in predicting the risk of IS.

**Results:**

The univariate logistic regression analysis (ULR) demonstrated that age, gender, TyG index, history of diabetes, history of hypertension, fasting blood glucose (FBG), systolic blood pressure(SBP), diastolic blood pressure(DBP), low-density lipoprotein cholesterol(LDL-C), cystatin C(Cys C), the presence or absence of carotid plaques, plaque location, carotid intima-media thickness(CIMT), the length and thickness of the largest plaque were significantly associated with IS (P < 0.05), while the P-values of triglycerides(TG), total cholesterol(TC), uric acid(UA) and carotid stenosis rate were greater than 0.05. The area under the ROC curve (AUC) of the TyG index for predicting IS was 0.645 (P < 0.001), indicating a certain predictive ability but relatively limited. The optimal cut-off value was 8.28, with a sensitivity of 0.83 and a specificity of 0.63 at this cut-off value. The stratified analysis based on quartiles of the TyG index revealed that as the TyG index increased, the prevalence of hypertension and diabetes, as well as multiple lipid and metabolic indicators, increased, and the characteristics of carotid plaques also changed. Multiple risk prediction models were constructed and analyzed by ROC curves. Model 1, which integrated traditional clinical indicators, TyG index and carotid ultrasound parameters, performed best (AUC = 0.932) (P < 0.001), while Model 16, which only included some carotid ultrasound indicators, had relatively low predictive efficacy (AUC = 0.750) (P < 0.001).

**Conclusion:**

This study confirms that the combination of TyG index and carotid ultrasound parameters is of great significance in predicting the risk of IS. The predictive ability of TyG index alone is limited, and Model 1 integrating multiple indicators has the best predictive effect and can provide a reference for clinical practice. However, due to the retrospective nature of this study and the limitations such as selection bias, small sample size and single-center, there are some discrepancies between some results and those of previous studies. Future studies need to conduct multi-center, large-sample studies and incorporate more factors to improve the model.

## Introduction

Ischemic stroke (IS) is a huge global health challenge (1), gravely threatening human health and quality of life. Insulin resistance (IR) plays a pivotal role as a key risk factor in the occurrence and development of IS (2, 3). In recent years, some studies have suggested that the triglyceride - glucose (TyG) index, as a promising surrogate marker for IR (4, 5), has a positive correlation with an increased risk of stroke (6, 7). However, most of the existing studies are confined to the relationships among clinical indicators, demographic indicators, and IS. Moreover, the effectiveness of the models in these studies varies. The NASCET trial has shown that < 50% lumen stenosis may be less likely to have a causal relationship with IS. Coronary pathology and intravascular ultrasound studies have suggested that < 50% lumen stenosis is caused by expansive remodeling, but these extensively remodeled lesions have the characteristics of vulnerable plaques and may be the source of plaque thrombosis and an under-recognized cause of IS (8). Carotid artery assessment is undergoing a shift from stenosis to plaque characteristics. The study by Kopczak et al. (9) demonstrated that complex plaques may lead to stroke even with a low degree of stenosis, pointing out that the research data from 20 - 30 years ago may not be in line with the modern situation. Improvements in treatment strategies and survival rates, as well as lifestyle changes, may have led to a shift in the underlying pathology of large artery strokes from ruptured to unruptured plaques. Other studies (10, 11) have found that the presence of carotid plaques and carotid intima - media thickness (CIMT) are associated with the risk of IS. Although several studies have investigated the relationships between the TyG index and carotid plaques with IS respectively, few studies have combined these two factors to observe and verify their predictive efficacy. This is of great significance for establishing a more effective predictive model of IS and for exploring the complex interaction mechanisms between clinical indicators such as the TyG index and different carotid ultrasound parameters. Therefore, this study aims to deeply analyze the roles of the TyG index and carotid ultrasound parameters (including the presence, size, location of carotid plaques and CIMT, etc.) in predicting the risk of IS by establishing multiple predictive models and comparing their statistical analysis results, hoping to be helpful for future in-depth research.

## Materials and methods

### Patient selection and criteria

This retrospective study included some adult IS patients and NIS patients who were diagnosed in our hospital from January 2020 to June 2024 by Magnetic Resonance Imaging (MRI). Inclusion Criteria: Onset time ≤ 72 h; Patients diagnosed by routine cranial MRI examination (symptoms of IS patients conform to the “Chinese Guidelines for the Diagnosis and Treatment of Acute Ischemic Stroke 2018”); Participants need to have complete clinical data, laboratory test results, and imaging data, etc. Exclusion Criteria: Hemorrhagic stroke(HS); Those with a history of brain tumors, severe liver and kidney dysfunction, thyroid nodules, or other organ tumors; Those with a history of infection and surgical trauma within 3 months (patients taking multiple drugs that may affect the body’s inflammation index).

### Data collection and definitions

In this study, the following indicators were collected for each patient: whether they have IS, age (in years), gender, history of hypertension, history of diabetes, systolic blood pressure (SBP) (mmHg), diastolic blood pressure (DBP) (mmHg), fasting blood glucose (FBG) (mg/dl), triglycerides (TG) (mg/dl), total cholesterol(TC) (mmol/l), low-density lipoprotein cholesterol (LDL-C) (mmol/l), uric acid (UA) (μmol/L), cystatin C (Cys C) (mg/l), presence or absence of carotid plaques, location of the largest carotid plaque, carotid intima-media thickness (CIMT) (mm), length diameter (mm) and thickness diameter (mm) of the largest carotid plaque, and carotid stenosis rate. The history of hypertension and diabetes was recorded based on the self-report of the patients or their family members. SBP and SDP were measured at admission, and the average of two measurements was taken. If the difference between the two measurements exceeded 5 mmHg, additional measurements were conducted. Hypertension was defined as SBP ≥ 140 mmHg or DBP ≥ 90 mmHg (12). FBG, TG, TC, LDL-C, UA, and Cys C were measured by collecting blood samples from patients after an overnight fast and processing them in the hospital’s central laboratory. The TyG index was calculated using the formula: ln [TG (mg/dl) × FBG(mg/dl)/2] (13).

Carotid ultrasound parameters were obtained by the doctors from the hospital’s ultrasound department through scanning with the GE LOGIQ E10 Color Doppler Ultrasound Diagnostic Instrument. CIMT is the mean of multiple measures of the maximum IMT of the near and far wall on both the left and right sides. The distance is from the first to the second leading edge of the echogenic line was regarded as CIMT of either side (14). Carotid plaque was defined as a focal structure encroaching into the arterial lumen of at least 0.5 mm; or 50% of the surrounding IMT value; or demonstrating a thickness > 1.5 mm, as measured from the media – adventitia interface to the intima – lumen interface (15). The doctor selects the longest and thickest parts of the plaque on multiple ultrasound planes where the plaque can be fully displayed to obtain the length and thickness diameters of the carotid plaque, and then selects the largest plaque based on the measurement results and determines its location. Degree of stenosis was expressed according to NAS- CET (North American Symptomatic Carotid Endarterectomy Trial) criteria (16). In a single study (17) reporting stenosis according to ECST (European Carotid Surgery Trial) criteria, values were transformed to NASCET values by using a published formula. Degree of stenosis was categorized into mild (< 50%), moderate (50% to 69%), or severe (70% to 99%) concordant with NASCET (18).

### Statistical analysis

In this study, statistical analysis was carried out using SPSS 26 software. Quantitative data with a normal distribution were presented as mean ± standard deviation ( $\bar{x}\pm SD$ ), and independent - sample t - tests were used for comparisons between groups. Data with a skewed - normal distribution were expressed as M (P25, P75), and Mann - Whitney U test (M-W U test) were employed for analysis. Categorical variables were described as frequencies (percentages), and chi - square tests (χ² test) or Fisher’s exact probability method (FEP) were used for inter - group comparisons.

The data were divided into an IS group and a NIS group. Inter - group differences in the characteristics of each group were analyzed, and the TyG index was grouped according to quartiles for comparing inter - group differences. Based on the results of the inter - group difference analysis and in combination with clinical reality, potentially related variables were included in the univariate logistic regression analysis (ULR) and the multivariate logistic regression analysis (MLR).

Multiple prediction models were established through MLR. These models included a model combining clinical indicators and carotid ultrasound parameters, a model combining only the TyG index and carotid ultrasound parameters, and a model with only carotid ultrasound parameters. Receiver operating characteristic (ROC) curve analysis was performed on each model. Finally, the cut - off value, sensitivity, and specificity of the TyG index for predicting IS were calculated. In all analyses, a significance level of P < 0.05 was considered statistically significant.

## Results

### Characteristics of the population with IS

A total of 244 participants were enrolled in this study, and they were divided into the IS group and the NIS group. There were significant differences between the two groups in terms of indicators such as age, gender, FBG, history of diabetes, history of hypertension, TyG index, SBP, DBP, LDL-C, Cys C, presence or absence of carotid plaques, location of the largest carotid plaque, CIMT, thickness of the largest carotid plaque, and carotid stenosis rate (P < 0.05). However, there were no significant differences between the two groups in TG,TC, UA, and length of the largest carotid plaque (P > 0.05). The detailed results are presented in **Table 1**.

**Table 1:** Baseline characteristics of patients with ischemic stroke.

Group	NIS (n=99)	IS (n=145)	P Value
Age	52±11	63±10	<0.001*
Gender			
Male	45 (45.5%)	91 (62.8%)	0.009*
Female	54 (54.5%)	54 (37.2%)	
FBG (mg/dl)	88.56 (82.62, 96.48)	104.40 (85.46, 142.56)	<0.001*
TG (mg/dl)	111.60 (82.37, 146.39)	115.15 (81.93, 164.24)	0.206
TyG	8.47 (8.15, 8.86)	8.69 (8.35, 9.25)	0.002*
Diabetes History			
Yes	18 (18.2%)	51 (35.2%)	0.004*
No	81 (81.8%)	94 (64.8%)	
Hypertension History			
Yes	27 (27.3%)	95 (65.5%)	<0.001*
No	72 (72.7%)	50 (34.5%)	
SBP (mmHg)	130±17	145±18	<0.001*
DBP (mmHg)	79±12	85±13	<0.001*
TC (mmol/l)	4.42 (3.68, 5.10)	4.49 (3.93, 5.20)	0.193
LDL-C (mmol/l)	2.51±0.81	2.73±0.83	0.042*
UA (μmol/L)	317±78	315±90	0.812
Cys C (mg/l)	0.75 (0.64, 0.84)	0.86 (0.74, 1.00)	<0.001*
Carotid Artery Plaque			
Yes	43 (43.4%)	122 (84.1%)	
No	56 (56.6%)	23 (15.9%)	<0.001*
Location of the largest plaque			
None	55 (55.6%)	23 (15.9%)	
Carotid Sinus	36 (36.4%)	96 (66.2%)	<0.001*
Carotid Body	8 (8.1%)	26 (17.9%)	
CIMT (mm)	1.0 (0.9,1.2)	1.2 (1.1,1.3)	<0.001*
Max Plaque Length (mm)	10.05 (5.83, 14.83)	12.40 (7.98, 16.90)	0.052
Max Plaque Thickness (mm)	2.10 (1.73, 2.65)	2.85 (2.20, 3.63)	<0.001*
SR			
Category I	99 (100%)	129 (89.0%)	<0.001*
Category II	0	16 (11.0%)	

TyG, Triglyceride Glucose Index; FBG, Fasting Blood Glucose; TG, Triglycerides; TC, Total Cholesterol; LDL-C, Low-Density Lipoprotein Cholesterol; UA, Uric Acid; Cys C, Cystatin C; SBP, Systolic Blood Pressure; DBP, Diastolic Blood Pressure; CIMT, Carotid Intima-Media Thickness; SR, Stenosis Rate; Category I, No stenosis or stenosis rate < 50%, Category II, Stenosis rate ≥ 50% or occlusion. *P < 0.05.

### Univariate logistic regression analysis of factors associated with IS

In the comparison of overall data between groups, although there were no significant differences in TG, TC, UA, and the length of the largest carotid plaque between the IS group and the NIS group, the results of numerous studies have indicated that they are risk factors for IS. Therefore, we chose to include all the above - mentioned variables in the ULR. The results showed that age, gender, TyG index, history of diabetes, history of hypertension, FBG, SBP, DBP, LDL - C, Cys C, presence or absence of carotid plaques, location of carotid plaques, CIMT, length and thickness of the largest carotid plaque were significantly associated with IS (P < 0.05), while the P - values of TG, TC, UA, and carotid stenosis rate were > 0.05. The detailed results are presented in **Table 2**.

**Table 2:** Univariate logistic regression analysis of factors associated with ischemic stroke.

Variable	B	OR	95%CI	P Value
Age	0.108	1.114	1.079, 1.149	<0.001*
Gender				
Male	0.704	2.022	1.203, 3.400	0.008*
Female			Reference	
FBG (mg/dl)	0.022	1.023	1.012, 1.033	<0.001*
TG (mg/dl)	0.004	1.004	0.999, 1.008	0.089
TyG	0.755	2.128	1.376, 3.291	0.001*
Diabetes History				
Yes	0.893	2.441	1.321, 4.512	0.004*
No			Reference	
Hypertension History				
Yes	1.415	4.117	2.366, 7.163	<0.001*
No			Reference	
SBP (mmHg)	0.053	1.055	1.036, 1.073	<0.001*
DBP (mmHg)	0.038	1.039	1.016, 1.062	0.001*
TC (mmol/l)	0.176	1.193	0.958, 1.486	0.116
LDL-C (mmol/l)	0.329	1.39	1.009, 1.914	0.044*
UA (μmol/L)	0.001	1	0.997, 1.003	0.811
Cys C (mg/l)	4.388	80.466	14.366, 450.710	<0.001*
Carotid Artery Plaque				
Yes	1.933	6.908	3.802, 12.550	<0.001*
No			Reference	
Location of the largest plaque				
None			Reference	
Carotid Sinus	1.853	6.377	3.433, 11.846	<0.001*
Carotid Bulb	2.05	7.772	3.067, 16.696	<0.001*
CIMT (mm)	5.532	252.745	45.411, 1406.716	<0.001*
Maximum Plaque Length (mm)	0.059	1.061	1.005, 1.120	0.032*
Maximum Plaque Thickness(mm)	1.009	2.744	1.647, 4.572	<0.001*
SR	5.363	0.005	0.000, 0.069	<0.001*
Category I			Reference	
Category II	20.938	>1000	0.000, >1000	0.998

TyG, Triglyceride Glucose Index; FBG, Fasting Blood Glucose; TG, Triglycerides; TC, Total Cholesterol; LDL-C, Low-Density Lipoprotein Cholesterol; UA, Uric Acid; Cys C, Cystatin C; SBP, Systolic Blood Pressure; DBP, Diastolic Blood Pressure; CIMT, Carotid Intima-Media Thickness; SR, Stenosis Rate; Category I, No stenosis or stenosis rate < 50%, Category II, Stenosis rate ≥ 50% or occlusion. *P < 0.05.

### ROC curve of TyG index predicting IS

**Figure 1:**
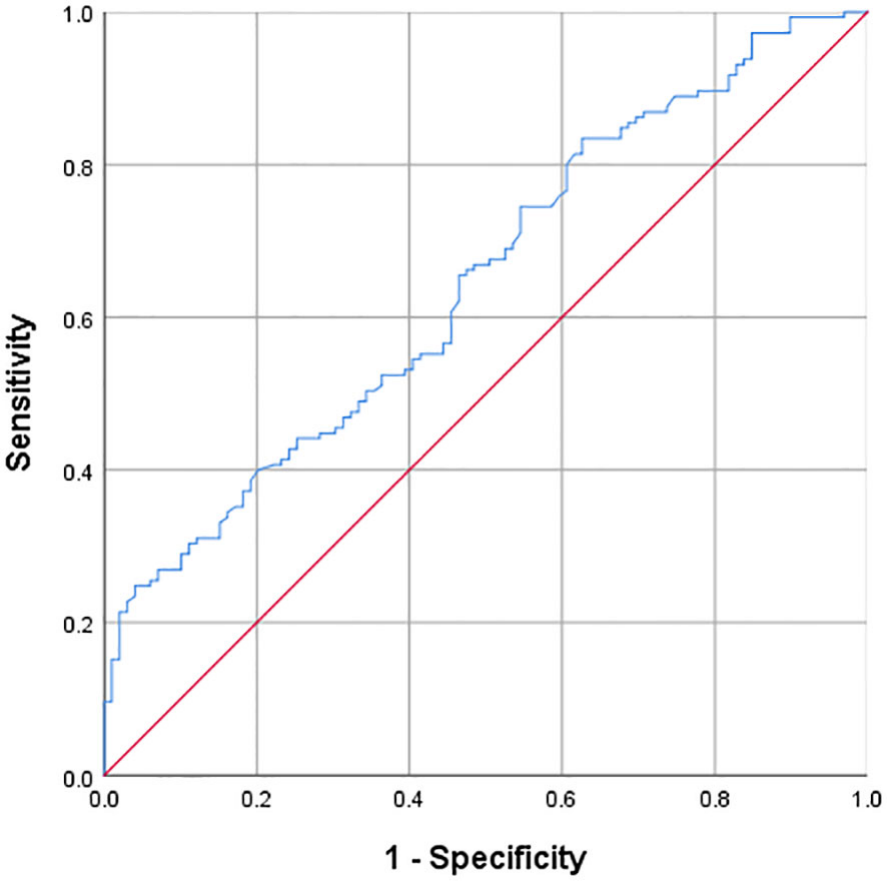
ROC curve of TyG index predicting ischemic stroke optimal cut-off value of TyG = 8.28; AUC = 0.645(P<0.001); Sensitivity = 0.83; Specificity = 0.63.

Through ULR, we found that the TyG index was significantly correlated with the risk of IS. Therefore, we explored the predictive value of the TyG index for IS by drawing the ROC curve. The area under the ROC curve (AUC) was 0.645 (P < 0.001), indicating that the TyG index has a certain predictive ability for IS, but the predictive ability is relatively limited. The optimal cut-off value of the TyG index for predicting IS was 8.28. At this cut-off value, the sensitivity was 0.83 and the specificity was 0.63. The detailed results are presented in **Figure 1**.

### Characteristics of the population stratified by the TyG index

**Table 3** presents the characteristics of the study population according to the quartiles of the TyG index. The ranges of the TyG index for Q1 - Q4 groups are < 8.27, 8.28 - 8.60, 8.61 - 8.99, and > 9.00, respectively. Among individuals with a higher TyG index level, there are more people with a history of hypertension and diabetes. The values of FBG, TG, SBP, DBP, TC, LDL - C, UA, and Cys C are higher. There are more people with carotid plaques, the largest carotid plaque is more likely to occur at the carotid sinus, and the length diameter of the largest carotid plaque is greater.

**Table 3:** Statistical analysis of various indicators based on quartiles of the TyG index.

Variable	Quartiles of the TyG Index				P Value
	Q1 (7.23-8.27) n=61	Q2 (8.28-8.60) n=61	Q3 (8.61-8.99) n=61	Q4 (9.00-10.86) n=61	
TyG	7.99 ± 0.23	8.44 ± 0.10	8.79 ± 0.11	9.57 ± 0.46	<0.001*
Age	56 ± 12	61 ± 11	59 ± 11	60 ± 12	0.07
Gender					
Male/Female	29/32	33/28	35/26	39/22	0.33
FBG (mg/dl)	83.83 ± 10.06	96.01 ± 23.68	104.95 ± 24.65	159.48 ± 68.81	<0.001*
TG (mg/dl)	72.39 ± 14.42	101.50 ± 22.83	131.47 ± 29.30	205.01 ± 81.44	<0.001*
Diabetes History					
Yes/No	1/60	8/53	19/42	41/20	<0.001*
Hypertension History					
Yes/No	18/43	31/30	29/32	37/24	0.010*
SBP (mmHg)	130 ± 19	140 ± 17	141 ± 20	145 ± 17	<0.001*
DBP (mmHg)	77 ± 13	82 ± 13	85 ± 12	85 ± 11	<0.001*
TC (mmol/l)	4.16 ± 1.11	4.55 ± 1.02	4.95 ± 1.35	4.76 ± 1.21	<0.001*
LDL-C (mmol/l)	2.34 ± 0.79	2.67 ± 0.74	2.90 ± 0.92	2.66 ± 0.77	<0.001*
UA (μmol/L)	289 ± 80	314 ± 63	327 ± 76	333 ± 110	0.020*
Cys C (mg/l)	0.75 ± 0.17	0.87 ± 0.23	0.87 ± 0.20	0.90 ± 0.32	<0.001*
Carotid Artery Plaque					
Yes/No	33/28	47/14	40/21	45/16	0.033*
Location of the largest plaque					
None/Carotid Sinus/ Carotid Bulb	28/31/2	14/34/13	20/30/11	16/37/8	0.021*
CIMT (mm)	1.12 ± 0.17	1.15 ± 0.19	1.16 ± 0.18	1.15 ± 0.19	0.69
Max Plaque Length (mm)	10.20 ± 5.52	12.32 ± 6.66	14.43 ± 9.47	15.13 ± 8.27	0.024*
Max Plaque Thickness (mm)	2.74 ± 1.10	2.81 ± 1.20	2.87 ± 1.26	2.86 ± 0.68	0.958
SR					
Category I/II	58/3	57/4	58/3	55/6	0.658

TyG, Triglyceride Glucose Index; FBG, Fasting Blood Glucose; TG, Triglycerides; TC, Total Cholesterol; LDL-C, Low-Density Lipoprotein Cholesterol; UA, Uric Acid; Cys C, Cystatin C; SBP, Systolic Blood Pressure; DBP, Diastolic Blood Pressure; CIMT, Carotid Intima-Media Thickness; SR, Stenosis Rate; Category I, No stenosis or stenosis rate < 50%, Category II, Stenosis rate ≥ 50% or occlusion. *P < 0.05.

### Risk prediction model for IS and ROC curves constructed by combining clinical indicators such as the TyG index with carotid ultrasound parameters

We adjusted the models by considering the significance of variables (P-values), the goodness of fit of the models, and the AUC based on traditional clinical indicators, the TyG index, and carotid ultrasound parameters, and constructed a total of seven models (Model1-4, Model6-8). Using the same method, we constructed one model (Model5) by including only traditional clinical indicators and carotid ultrasound parameters without the TyG index. Four models (Model9-11, Model13) were constructed by combining the TyG index and carotid ultrasound parameters. Four models (Model12, Model14-16) were constructed using only carotid ultrasound parameters. The AUC values of Model 1 to Model 16 are 0.932, 0.930, 0.929, 0.929, 0.901, 0.923, 0.922, 0.921, 0.862, 0.800, 0.800, 0.799, 0.839, 0.819, 0.819, and 0.750 respectively. (P<0.001) The factors included in the models are shown in **Tables 4** and **5**.

**Table 4:** Predictive models of ischemic stroke risk constructed by combining clinical indicators and carotid ultrasound parameters.

Variable	Model 1	Model 2	Model 3	Model 4	Model 5	Model 6	Model 7	Model 8
	P Value	P Value	P Value	P Value	P Value	P Value	P Value	P Value
TyG	<0.001*	<0.001*	<0.001*	<0.001*	——	<0.001*	0.001*	<0.001*
Age	l0.381	<0.001*	<0.001*	<0.001*	<0.001*	<0.001*	<0.001*	<0.001*
Gender (Male)	0.076	0.014*	0.032*	0.030*	0.283	0.017*	0.018*	0.003*
FBG (mg/dl)	0.004*	<0.001*	<0.001*	<0.001*	0.015*	<0.001*	<0.001*	<0.001*
TG (mg/dl)	0.002*	<0.001*	<0.001*	<0.001*	0.005*	<0.001*	<0.001*	<0.001*
Diabetes History (Yes)	0.584	——	——	——	——	——	——	——
Hypertension History (Yes)	0.407	0.246	0.368	0.361	<0.001*	0.587	——	0.566
SBP (mmHg)	0.037*	0.023*	0.024*	0.023*	0.325	0.003*	<0.001*	0.004*
DBP (mmHg)	0.358	0.027*	0.015*	0.015*	0.231	0.032*	0.013*	0.018*
TC (mmol/l)	0.298	0.881	0.757	——	——	——	——	——
LDL-C (mmol/l)	0.537	0.276	0.21	0.086	0.901	0.138	0.136	0.168
Cys C (mg/l)	0.032*	0.020*	0.026*	0.025*	0.124	0.031*	0.031*	0.027*
Carotid Artery Plaque (Yes)	0.437	0.265	0.301	0.307	<0.001*	——	——	——
Location of the largest plaque								
None	0.301	0.31	0.358	0.369	<0.001*	——	——	——
Carotid Sinus	<0.001*	0.129	0.152	0.158	<0.001*			
Carotid Bulb	0.301	0.825	0.987	0.988	0.825			
CIMT (mm)	0.068	<0.001*	<0.001*	<0.001*	<0.001*	<0.001*	<0.001*	0.001*
Max Plaque Length (mm)	0.333	0.061	——	——	——	——	——	——
Max Plaque Thickness (mm)	0.007*	<0.001*	<0.001*	0.001*	0.002*	0.088	0.086	——

TyG, Triglyceride Glucose Index; FBG, Fasting Blood Glucose; TG, Triglycerides; TC, Total Cholesterol; LDL-C, Low-Density Lipoprotein Cholesterol; UA, Uric Acid; Cys C, Cystatin C; SBP, Systolic Blood Pressure; DBP, Diastolic Blood Pressure; CIMT, Carotid Intima-Media Thickness;——, The factors not included in the model. *P < 0.05.

**Table 5:** Predictive models of ischemic stroke risk constructed by combining TyG index and carotid ultrasound parameters.

Variable	Model 9	Model 10	Model 11	Model 12	Model 13	Model 14	Model 15	Model 16
	P Value	P Value	P Value	P Value	P Value	P Value	P Value	P Value
TyG	0.053	<0.001*	<0.001*	——	0.026*	——	——	——
Age	<0.001*	<0.001*	<0.001*	0.157	——	——	——	——
Gender (Male)	0.25	0.607	0.609	0.484	——	——	——	——
Carotid Artery Plaque (Yes)	<0.001*	——	——	——	<0.001*	<0.001*	<0.001*	0.166
Location of the largest plaque								
None	<0.001*	0.544	0.535	0.001*	<0.001*	<0.001*	<0.001*	——
Carotid Sinus	<0.001*	0.949	0.958	<0.001*	<0.001*	<0.001*	<0.001*	——
Carotid Bulb	0.926	0.337	0.324	0.005*	0.348	0.473	0.554	——
CIMT (mm)	<0.001*	0.016*	0.016*	0.777	<0.001*	<0.001*	<0.001*	0.005*
Max Plaque Length (mm)	0.574	0.954	——	——	0.428	0.601	——	——
Max Plaque Thickness (mm)	<0.001*	0.001*	<0.001*	0.003*	<0.001*	<0.001*	<0.001*	<0.001*

TyG, Triglyceride Glucose Index; FBG, Fasting Blood Glucose; TG, Triglycerides; TC, Total Cholesterol; LDL-C, Low-Density Lipoprotein Cholesterol; UA, Uric Acid; Cys C, Cystatin C; SBP, Systolic Blood Pressure; DBP, Diastolic Blood Pressure; CIMT, Carotid Intima-Media Thickness; ——, The factors not included in the model. *P < 0.05.

**Figure 2:**
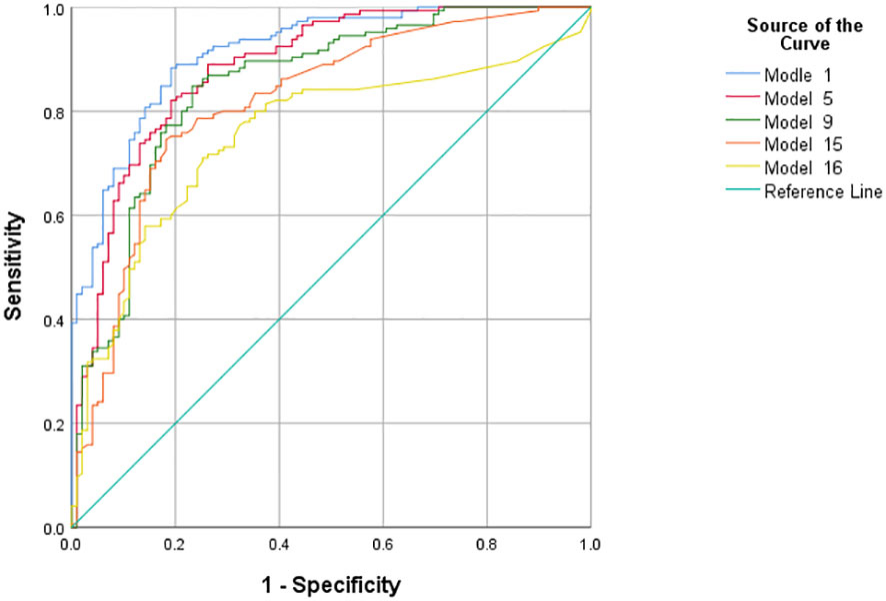
ROC curves of models predicting ischemic stroke.

Then, we used the predicted probabilities obtained from each model through MLR to draw ROC curves and calculate the AUC to evaluate the predictive performance of these models. We compared the predictive performance of five models, namely the models with the best predictive efficacy obtained after inclusion according to the above four inclusion mechanisms (Model1, 5, 9, 15), and the model (Model16) constructed by including only three indicators of carotid ultrasound parameters: the presence or absence of carotid plaques, CIMT, and the maximum thickness of carotid plaques, through ROC curves (**Figure 2**). The results showed that the AUC of Model 1 was 0.932, the AUC of Model 5 was 0.901, the AUC of Model 9 was 0.862, the AUC of Model 15 was 0.819, and the AUC of Model 16 was 0.750. All P-values were less than 0.001.

## Discussion

This study focused on the role of the combination of TyG index and carotid ultrasound parameters in predicting the risk of IS. Through a retrospective analysis of 244 patients, a series of significant results were obtained, which had multiple associations and differences with previous studies. In terms of clinical indicators, this study found that there were significant differences between the IS group and the NIS group in age, gender, FBG, diabetes history, hypertension history, TyG index, SBP, DBP, LDL - C, the presence or absence of carotid plaques, the location of the largest carotid plaque, CIMT, the thickness of the largest carotid plaque, and the carotid stenosis rate. This is highly consistent with many previous research (19, 20) results and strongly supports the crucial role of these factors in the pathogenesis of IS. For example, numerous studies (21, 22) have clearly identified that age growth, hypertension, diabetes, and dyslipidemia are important risk factors for IS. This study re-verified the close relationship between these factors and IS, further strengthening the necessity of comprehensively evaluating these indicators in clinical practice to identify high-risk populations of IS at an early stage. Meanwhile, the significant difference in the TyG index between the two groups, as a potential surrogate marker of IR, is also in line with the views on the association between metabolic factors and stroke risk in previous studies (7, 23), further highlighting its important position in the pathogenesis of IS. Although TG, TC, UA, and the length of the largest carotid plaque did not show significant differences, considering the clinical practice, the above indicators may still be risk factors for IS (20, 24–26).

Then, some studies have found that changes in creatinine levels may affect the TyG index (27, 28). From a physiological mechanism perspective, creatinine is a product of muscle metabolism and is mainly excreted through the kidneys. Abnormal renal function may lead to an increase in creatinine levels, and kidney dysfunction may also affect the body’s metabolic state, thereby indirectly influencing the metabolic relationship between TC and FBG reflected by the TyG index. In recent years, multiple research findings have suggested that Cys C has significant advantages over the traditional creatinine level in the assessment of renal function (29, 30). Therefore, this study innovatively included Cys C in the research variable category for the first time. After data analysis and statistical testing, the results strongly indicate that Cys C is highly likely to be an independent risk factor for IS and shows important potential value in the research fields of IS pathogenesis and risk prediction.

Through ROC curve analysis, this study obtained that the AUC of the TyG index for predicting IS was 0.645, indicating a certain predictive ability but relatively limited. The optimal cut-off value was 8.28, and at this cut-off value, the sensitivity was 0.83 and the specificity was 0.63. Compared with other related studies, the results of this study are within a certain reasonable range numerically, but it also shows the necessity of finding a more efficient prediction model. Some previous studies have also reported the predictive value of the TyG index for IS, but there are certain differences in AUC and cut-off values, which may be attributed to factors such as the region, race, and sample size of the study population. For example, the population included in some studies may have a specific lifestyle, which affects the degree of association between the TyG index and IS. This study further suggests that relying solely on the TyG index for IS prediction has limitations in clinical application and requires the combination of other indicators to construct a comprehensive model. The stratified analysis based on the quartiles of the TyG index revealed the trend of its association with other risk factors. As the TyG index increased, the prevalence of hypertension and diabetes, as well as multiple lipid and metabolic indicators, increased, and the characteristics of carotid plaques also changed (7, 31). This is consistent with the research conclusions on the relationship between metabolic disorders and vascular lesions in the past, providing strong evidence for in-depth understanding of the internal relationship between them and also providing new ideas for clinical stratified management, that is, considering incorporating the TyG index into the comprehensive assessment system to implement more targeted interventions for patients at different risk levels.

In **Table 4**, variables such as TyG index, age, diabetes history, hypertension history, SBP, DBP, LDL - C, Cys C, carotid plaque, CIMT, and maximum plaque thickness had P values less than 0.05 in multiple models, indicating that these variables were significantly associated with IS and had a strong basis for inclusion in the prediction model. For example, in Model 1, the significance levels of variables such as TyG index (P < 0.001) and age (P = 0.381) showed that they played an important role in predicting the risk of IS in the comprehensive model. The OR value (1.114) and 95% CI (1.079, 1.149) of age indicated that the risk of IS increased with age growth, and its role in the model could not be ignored. Some variables such as gender had P values close to or slightly greater than 0.05 in some models (e.g., P = 0.076 in Model 1). Although not highly significant, they may still have a certain impact on IS (32). In further research or model optimization, they can be considered to be retained or their interactions with other variables can be analyzed in depth. In **Table 5**, variables such as the TyG index, carotid plaque, and CIMT were significant in multiple models, which once again emphasized their crucial positions in the prediction model. Moreover, through the comparison of the performance of Model 15 and Model 16, it was concluded that the variable of carotid plaque location also played a certain positive role in IS prediction.

Multiple models were constructed and the predictive performance of different combinations of models was compared. The results showed that Model 1 (including TyG index and multiple parameters) performed best (AUC = 0.932), while Model 16 (including only some carotid ultrasound indicators) had lower predictive efficacy (AUC = 0.750) and poorer model fit. Model 1 more comprehensively reflects the metabolic and vascular pathological states of patients, and its higher AUC reflects the good discriminative ability of the synergistic effect of various factors on the risk of IS, which is expected to provide doctors with more accurate risk assessment references in clinical applications and assist in formulating prevention and treatment plans. In contrast, Model 16 is limited by variables only considering local carotid characteristics and lacks consideration of systemic factors such as metabolism, resulting in limited predictive ability, which further confirms the importance of constructing models by combining multiple types of indicators.

As a retrospective study, this study inevitably has certain limitations. For example, the relatively limited sample size may not accurately reflect the relationship between some variables and IS. In addition, this is a retrospective single-center study based on the existing case data in the hospital. Although strict inclusion and exclusion criteria were set, sufficient sample data with a high degree of carotid stenosis were not collected, resulting in the failure to reveal the correlation between the carotid stenosis rate and IS risk. Finally, although this study compensates for the possible misjudgment of results due to the small sample size by presenting the P values and AUC values of models with different variable compositions, if multi-center and large-sample studies can be carried out in future research, combined with more scientific and rigorous statistical analysis methods, the IS risk prediction model can be further improved and its clinical application value can be enhanced.

## Conclusion

To summarize, our study demonstrates that the combination of clinical indicators such as the TyG index and multiple carotid ultrasound parameters can better predict the risk of IS. These findings suggest that future research should actively incorporate imaging data such as ultrasound into the construction of IS risk prediction models, as it may contribute to the early identification and prediction of IS. It is valuable to further investigate the mechanisms of various carotid ultrasound parameters in IS risk prediction and how to develop the optimal IS prediction model.

## Data Availability

The original contributions presented in the study are included in the article. Further inquiries can be directed to the corresponding author.

## References

[1] Roth GA Mensah GA Johnson CO Addolorato G Ammirati E Baddour LM. Global burden of cardiovascular diseases and risk factors, 1990-2019: update from the GBD 2019 study. J Am Coll Cardiol. (2020) 76:2982–3021. doi: 10.1016/j.jacc.2020.11.010 33309175 PMC7755038

[2] Bornfeldt KE Tabas I. Insulin resistance, hyperglycemia, and atherosclerosis. Cell Metab. (2011) 14:575–85. doi: 10.1016/j.cmet.2011.07.015 PMC3217209 22055501

[3] Kernan WN Inzucchi SE Viscoli CM Brass LM Bravata DM Horwitz RI. Insulin resistance and risk for stroke. Neurology. (2002) 59:809–15. doi: 10.1212/wnl.59.6.809 12349850

[4] Hong S Han K Park CY. The triglyceride glucose index is a simple and low-cost marker associated with atherosclerotic cardiovascular disease: a population-based study. BMC Med. (2020) 18:361. doi: 10.1186/s12916-020-01824-2 33234146 PMC7687762

[5] Guerrero-Romero F Villalobos-Molina R Jiménez-Flores JR Simental-Mendia LE Méndez-Cruz R Murguía-Romero M. Fasting triglycerides and glucose index as a diagnostic test for insulin resistance in young adults. Arch Med Res. (2016) 47:382–7. doi: 10.1016/j.arcmed.2016.08.012 27751372

[6] Shi W Xing L Jing L Tian Y Yan H Sun Q. Value of triglyceride-glucose index for the estimation of ischemic stroke risk: Insights from a general population. Nutr Metab Cardiovasc Dis. (2020) 30:245–53. doi: 10.1016/j.numecd.2019.09.015 31744716

[7] Wang A Wang G Liu Q Zuo Y Chen S Tao B. Triglyceride-glucose index and the risk of stroke and its subtypes in the general population: an 11-year follow-up. Cardiovasc Diabetol. (2021) 20:46. doi: 10.1186/s12933-021-01238-1 33602208 PMC7893902

[8] Willey JZ Pasterkamp G. The role of the vulnerable carotid plaque in embolic stroke of unknown source. J Am Coll Cardiol. (2022) 79:2200–2. doi: 10.1016/j.jacc.2022.04.004 35523660

[9] Kramer CM Treiman GS. Vulnerable plaque in carotid arteries without “Significant” Stenosis. J Am Coll Cardiol. (2020) 76:2223–5. doi: 10.1016/j.jacc.2020.09.531 33153581

[10] Helmersson-Karlqvist J Lipcsey M Ärnlöv J Bell M Ravn B Dardashti A. Addition of cystatin C predicts cardiovascular death better than creatinine in intensive care. Heart. (2022) 108:279–84. doi: 10.1136/heartjnl-2020-318860 PMC8819658 33795382

[11] Alizargar J Bai CH. Comparison of carotid ultrasound indices and the triglyceride glucose index in hypertensive and normotensive community-dwelling individuals: A case control study for evaluating atherosclerosis. Medicina (Kaunas). (2018) 54:71. doi: 10.3390/medicina54050071 30344302 PMC6262598

[12] Parish S Arnold M Clarke R Du H Wan E Kurmi O. Assessment of the role of carotid atherosclerosis in the association between major cardiovascular risk factors and ischemic stroke subtypes. JAMA Netw Open. (2019) 2:e194873. doi: 10.1001/jamanetworkopen.2019.4873 31150080 PMC6547114

[13] Tian X Zuo Y Chen S Liu Q Tao B Wu S. Triglyceride-glucose index is associated with the risk of myocardial infarction: an 11-year prospective study in the Kailuan cohort. Cardiovasc Diabetol. (2021) 20:19. doi: 10.1186/s12933-020-01210-5 33435964 PMC7802156

[14] Jia X Zhu Y Qi Y Zheng R Lin L Hu C. Association between triglyceride glucose index and carotid intima-media thickness in obese and nonobese adults. J Diabetes. (2022) 14:596–605. doi: 10.1111/1753-0407.13312 36071605 PMC9512765

[15] Stein JH Korcarz CE Hurst RT Lonn E Kendall CB Mohler ER. Use of carotid ultrasound to identify subclinical vascular disease and evaluate cardiovascular disease risk: a consensus statement from the American Society of Echocardiography Carotid Intima-Media Thickness Task Force. Endorsed by the Society for Vascular Medicine. J Am Soc Echocardiogr. (2008) 21:93–111. doi: 10.1016/j.echo.2007.11.011 18261694

[16] Schindler A Schinner R Altaf N Hosseini AA Simpson RJ Esposito-Bauer L. Prediction of stroke risk by detection of hemorrhage in carotid plaques. JACC: Cardiovasc Imaging. (2020) 13:395–406. doi: 10.1016/j.jcmg.2019.03.028 31202755

[17] Esposito-Bauer L Saam T Ghodrati I Pelisek J Heider P Bauer M. MRI plaque imaging detects carotid plaques with a high risk for future cerebrovascular events in asymptomatic patients. PloS One. (2013) 8:e67927. doi: 10.1371/journal.pone.0067927 23894291 PMC3722215

[18] Rothwell PM Gibson RJ Slattery J Sellar RJ Warlow CP. Equivalence of measurements of carotid stenosis. A comparison of three methods on 1001 angiograms. European Carotid Surgery Trialists’ Collaborative Group. Stroke. (1994) 25:2435–9. doi: 10.1161/01.str.25.12.2435 7974586

[19] Zhang N Chi X Zhou Z Song Y Li S Xu J. Triglyceride-glucose index is associated with a higher risk of stroke in a hypertensive population. Cardiovasc Diabetol. (2023) 22:346. doi: 10.1186/s12933-023-02082-1 38093283 PMC10720217

[20] Wang X Feng B Huang Z Cai Z Yu X Chen Z. Relationship of cumulative exposure to the triglyceride-glucose index with ischemic stroke: a 9-year prospective study in the Kailuan cohort. Cardiovasc Diabetol. (2022) 21:66. doi: 10.1186/s12933-022-01510-y 35505313 PMC9066788

[21] Ma X Han Y Jiang L Li M. Triglyceride-glucose index and the prognosis of patients with acute ischemic stroke: A meta-analysis. Horm Metab Res. (2022) 54:361–70. doi: 10.1055/a-1853-9889 35697045

[22] Zhao Y Sun H Zhang W Xi Y Shi X Yang Y. Elevated triglyceride-glucose index predicts risk of incident ischaemic stroke: The Rural Chinese cohort study. Diabetes Metab. (2021) 47:101246. doi: 10.1016/j.diabet.2021.101246 33722769

[23] Yang Y Huang X Wang Y Leng L Xu J Feng L. The impact of triglyceride-glucose index on ischemic stroke: a systematic review and meta-analysis. Cardiovasc Diabetol. (2023) 22:2. doi: 10.1186/s12933-022-01732-0 36609319 PMC9825038

[24] Liu D Yang K Gu H Li Z Wang Y Wang Y. Predictive effect of triglyceride-glucose index on clinical events in patients with acute ischemic stroke and type 2 diabetes mellitus. Cardiovasc Diabetol. (2022) 21:280. doi: 10.1186/s12933-022-01704-4 36510223 PMC9743618

[25] Huang Z Ding X Yue Q Wang X Chen Z Cai Z. Triglyceride-glucose index trajectory and stroke incidence in patients with hypertension: a prospective cohort study. Cardiovasc Diabetol. (2022) 21:141. doi: 10.1186/s12933-022-01577-7 35897017 PMC9331781

[26] Wang X Liu Q Wang T Tian W Chen X Zhang J. Triglyceride-glucose index and the risk of stroke in American adults: findings from the atherosclerosis risk in communities study. Diabetol Metab Syndr. (2023) 15:187. doi: 10.1186/s13098-023-01161-3 37723527 PMC10507886

[27] Lv L Zhou Y Chen X Gong L Wu J Luo W. Relationship between the tyG index and diabetic kidney disease in patients with type-2 diabetes mellitus. Diabetes Metab Syndr Obes. (2021) 14:3299–306. doi: 10.2147/DMSO.S318255 PMC8296712 34305401

[28] Li L Xu Z Jiang L Zhuang L Huang J Liu D. Triglyceride-glucose index and its correlates: associations with serum creatinine and estimated glomerular filtration rate in a cross-sectional study from CHARLS 2011-2015. Metab Syndr Relat Disord. (2024) 22:179–89. doi: 10.1089/met.2023.0188 38133543

[29] Carrero JJ Fu EL Sang Y Ballew S Evans M Elinder CG. Discordances between creatinine- and cystatin C-based estimated GFR and adverse clinical outcomes in routine clinical practice. Am J Kidney Dis. (2023) 82:534–42. doi: 10.1053/j.ajkd.2023.04.002 37354936

[30] Spencer S Desborough R Bhandari S. Should cystatin C eGFR become routine clinical practice? Biomolecules. (2023) 13:1075. doi: 10.3390/biom13071075 37509111 PMC10377068

[31] Hoshino T Mizuno T Ishizuka K Takahashi S Arai S Toi S. Triglyceride-glucose index as a prognostic marker after ischemic stroke or transient ischemic attack: a prospective observational study. Cardiovasc Diabetol. (2022) 21:264. doi: 10.1186/s12933-022-01695-2 36451149 PMC9714168

[32] Cao JJ Thach C Manolio TA Psaty BM Kuller LH Chaves PH. C-reactive protein, carotid intima-media thickness, and incidence of ischemic stroke in the elderly: the cardiovascular health study. Circulation. (2003) 108:166–70. doi: 10.1161/01.CIR.0000079160.07364.6A 12821545

